# Book Reviews

**Published:** 1893-12

**Authors:** 


### Book Reviews.

A TEXT-BOOK OF OPHTHALMOLOGY. By WILLIAM F. NORRIS, M. D., Professor of Ophthalmology in the University of Pennsylvania, and CHARLES A. OLIVER, M. D., Surgeon to the Wills Eye Hospital, Philadelphia. In one very handsome octavo volume of 641 pages, with 357 engravings and five colored plates. Cloth, $5.00; leather, $6.00. Philadelphia: Lea Brothers & Co. 1893.

Advances in ophthalmology have been so rapid during the past decade, that it is not surprising that a number of text-books on the subject should have appeared of late. One of the most recent, as well as one of the best of these, has recently been presented to the profession by Prof. William F. Norris, of the University of Pennsylvania, together with Dr. Charles A. Oliver, and is published by the well-known firm of Lea Brothers & Co.

It is a book of 600 pages or more, and the many students who have been under Prof. Norris, and others as well, cannot but regard with interest any such work coming from his hands. Although it is issued as a text-book of ophthalmology, one soon perceives from the style that the lectures which he delivered to the students of the University have here been formulated, and this didactic method tends at first to produce an unfavorable impression, except to those who have been accustomed to listen to the senior author's lectures. One soon becomes accustomed to this, however, and to the general practitioner, as also to the specialist well versed in the subject, this style becomes rather attractive than otherwise.

The book very properly opens with a chapter on the Embryology of the Eye, a subject usually too much neglected, and in a few pages a good outline of that branch of the subject is sketched. The portions which treat of optics, the usual *bete-noir* of the student, have here been handled in a simple and systematic manner. With the teacher's instinct for clearness, the author begins by tracing the ray of light as it passes straight through a meridium with parallel sides, then shows how it is refracted by a prism, next by a combination of prisms, and finally, while the student imagines that he is dealing with elementary facts, he finds he has familiarized himself with the principles upon which the most complicated laws of optics depend.

In a chapter on the Examination of the Eye, the student is instructed to follow carefully a certain routine which is described, and although the directions given for this are rather detailed, and not at all times clear, the lesson of thoroughness is so taught that one cannot fail to be impressed by the great advantage to be derived from it in this branch above all others. The value of careful observations and accuracy in recording the detail of data found, is also most wisely insisted upon. It is, however, particularly in the chapters which relate to the more recent advances of ophthalmology, that this text-book is especially admirable. Inasmuch as the fundus reflex test (the shadow test) has of late been receiving so much attention, it is gratifying to find a whole chapter devoted to this method of measuring the refraction. The text is illustrated here with frequent diagrams, which give the reader a clear idea as to the optical principles upon which this method of examination depends.

The more recent methods of measuring the cornea are also given, and an illustration and short description is included of the

Javal–Schiotz instrument, now so common in the office of ophthalmologists.

In the chapter on the Muscles, the most recent views are presented, and it is worthy of note that the authors retain the nomenclature which may be considered thoroughly American.

It is with satisfaction that we observe in this connection a clearness of statement which tends to lessen the confusion, usually so manifest, concerning abnormal conditions of the ocular muscles, and it is also gratifying to observe that the writers do not allow themselves to be influenced by the vagaries which are of late too common in regard to indiscriminate tenotomies. The book is full of illustrations which, although taken almost entirely from other authors, are yet so well chosen as to elucidate at a glance any difficulties in the text, while a complete index brings any desired portion of the book quickly within reach. The typographical finish is admirable, and reflects credit upon the publishers, who will probably find themselves well repaid in this effort to offer to the profession so excellent and complete a text-book of ophthalmology. L. H.

---

MODERN GYNECOLOGY : A Treatise on Diseases of Women, comprising the results of the latest investigations and treatment in this branch of medical science. By CHARLES H. BUSHONG, M. D., Assistant Gynecologist to the Demilt Dispensary, New York ; formerly Attending Physician to the Northern Dispensary, and Assistant to the Vanderbilt Clinic, College of Physicians and Surgeons, New York. Illustrated. Octavo volume, pp. 380. Price, $2.75. New York : E. B. Treat, 5 Cooper Union. 1893.

Added to the large number of treatises on the diseases of women we here find another that is presented for the favor of the profession. It seems to be the purpose of this author to instruct the general practitioner (we presume he means of medicine), otherwise the family physician, in the science and art of gynecology. He asserts in his introduction that when this class of physicians seeks to inform themselves on this subject, they are generally offered books as large as the volumes from which they studied the entire subject of the practice of medicine. He says, further, that it is not surprising that busy men should feel their time inadequate for mastering so large a subject.

The fact is, in our observation, that all physicians are busy men, and we do not expect them to master every possible subject or branch of medical art. If they equip themselves so fairly well as to be able to diagnosticate the common ailments of womankind, so as to be able to recommend when necessary the proper specialist, that is about all that they will be required to do in the department of gynecology. It is utterly impossible for the general practitioner of medicine to acquire that dexterity in the use of instruments and in the delicacy of touch, necessary to diagnosticate and treat successfully the more intricate maladies pertaining to women.

However, the book before us is a fair treatise on the subject for a small one, and undoubtedly will command a large sale from the fact that the subjects are so arranged as to be easily accessible, and the mechanical part of the book is well-nigh perfect. Some of the illustrations, however, are not calculated to give a very clear idea of the subjects to which they pertain. This is the case especially with a number of those figures that are "made from photographs expressly for this work." For instance, it is difficult to see what information can be obtained beyond what is already possessed by an average person of intelligence, from figure 1, on page 21. Figure 19, on page 43, borrowed from Savage, has done duty so long that we fear it is pretty well worn out. Figure 23, on page 47, Cleveland's speculum in position, is not a very satisfactory exposition of the subject.

If our author expects to enlighten the family physician on the use of the uterine sound, he had better warn him against its dangers in a few brief paragraphs, and not devote several pages to its description and its application to the redressing of uterine displacements. Jennison's repositor, or uterine sound, is illustrated on page 269, figure 80, but what possible use this instrument can be made to serve, it is difficult for us to understand. It seems to be high time that authors should only illustrate useful instruments in their books, and we unhesitatingly declare this to be useless beyond all measure. Some of the figures have been made to do double duty, such as figure 78, on page 262, and figure 88, on page 281. The former is Dr. Campbell's old drawing of the genu-pectoral posture, and has done duty in nearly every gynecological treatise since 1873.

The peculiarities of this work may be accounted for by the fact that the author is one of the junior members of the profession, and no doubt will improve his treatise by illustrations and additions as experience teaches him to differentiate as to the value of procedures and instruments.

Supplement to the Reference Handbook of the Medical Sciences. By various writers. Illustrated by chromo-lithographs and fine wood-engravings. Edited by Albert H. Buck, M. D., New York City. Volume IX. Imperial octavo, 1084 pages. Cloth, price, $6.00 ; sheep, price, $7.00 ; half morocco, price, $8.00. New York : William Wood & Company.

The editor announces in the preface of this work that this volume is the outcome of a decision to bring the reference handbook of the medical sciences fully up to date. He says that it was at first contemplated to revise the volume of the work and issue a new edition; but, as this would practically make obsolete a great number of sets of the handbook now in the libraries of physicians all over the world, it was considered preferable to publish a supplementary volume, which could be added to those already issued, completing rather than destroying the original edition, and at the same time enabling present possessors of the work to obtain the new matter at a comparatively small cost. This has been done at great expense and after an unusual outlay of money; for it soon became evident that the volume could not be kept within the limits set beforehand, and the publishers were appealed to, who generously came to the rescue, and authorized the editor to publish all the extra material in its entirety, notwithstanding the great increase in cost to which the Messrs. Wood would thereby be subjected ; hence this volume far exceeds any of its predecessors in the number of pages which it contains. It is well illustrated in many parts, and its type, presswork, paper, and binding are beyond criticism. It contains a vast amount of information that can nowhere else be found, and fulfils the place to which it is assigned—namely, that of a reference handbook—to a more complete extent than any similarly named work.

To indicate something of its scope, let us select at random one title—namely, that of Influenza. This title is written by Dr. W. J. Conklin, of Dayton, Ohio, and begins with its definition. Dr. Conklin states that influenza is an acute, self-limited, infectious fever, occurring in widely distributed epidemics, and characterized by catarrhal inflammation of the respiratory and gastro-intestinal mucosa, by profound nervous disturbances and by extreme debility. Its synonyms are then given—towit : febris catarrhalis, epidemic catarrhal fever, la grippe, grip, tac, horion, la dandoziep, epidemischer husten, epidemischer schnupfen, schafhusten, blitz catarrh, mödefieber, mal russe, snufsjuka (Swedish), qual-tong (Chinese).

A page is devoted to a historical sketch of the disease, which is most interesting reading; then the etiology of the malady is described in another page; next its morbid anatomy is considered in a few short paragraphs; then its symptoms are detailed at much length, and, afterward, its complications and sequelæ are described; then its prognosis, diagnosis, and, finally, its treatment are considered. A bibliographical index is added, which completes the article, the whole requiring about six pages of space.

This is a sample of the thoroughness with which the articles are prepared for this work, and each article is signed by the author, who thus becomes responsible for any inaccuracies of statement.

It is a valuable addition to the literature of the period, and will easily find a place on the book-shelves of progressive physcians.

---

THE MEDICAL NEWS VISITING LIST FOR 1894. Weekly (dated, for thirty patients); Monthly (undated, for 120 patients per month); Perpetual (undated, for thirty patients weekly per year); and Perpetual (undated, for sixty patients weekly per year). The first three styles contain thirty-two pages of data and 176 pages of blanks. The Sixty-Patient Perpetual consists of 256 pages of blanks. Each style in one wallet-shaped book, pocket, pencil, rubber, and catheter-scale, etc. Seal grain leather, $1.25. Philadelphia: Lea Brothers & Co. 1893.

This regular annual is one of the most compact and useful of all the visiting lists in the market. It is neatly bound in seal morocco, with gilt edges and thumb-index. It contains, besides the usual record of practice, which in the book before us is arranged for thirty patients per week, the following:

How to keep accounts; how to keep a visiting list; dentition; to find the day of confinement; thermometric scales; ordinary weights and measures; the metric system; examination of the urine; important incompatibles; artificial respiration (Sylvester's method); list of poisons and antidotes; table of doses of remedies most frequently administered; and therapeutic reminders. In addition there is a clinical record, a consultation record, and a record of obstetrical engagements, a practice and vaccination list, a death register, a list of addresses, and a cash account.

It is published in four styles, namely, weekly, dated for thirty patients; monthly, undated, for 120 patients per month; perpetual, undated, for thirty patients per week per year; perpetual, undated, sixty patients per week per year (without text). The first three styles contain thirty-two pages of text and 176 pages of blanks, and the sixty-patient style contains 256 pages of blanks. It is made in wallet size, with flexible leather cover, and contains a pocket with pencil and catheter scale. Price, in any style, $1.25 ; to subscribers of the *News*, 75c. Thumb-letter index for rapid use of visiting list, twenty-five cents extra.

---

REACTIONS. A Selection of Organic Chemical Preparations Important to Pharmacy in Regard to Their Behavior to Commonly Used Reagents. By F. A. FLUECKIGER, Ph. D., M. D. Translated, revised, and enlarged by J. B. Nagelvoort, Analytical Chemist to the Pharmacal Chemical Laboratory of Parke, Davis & Co. Authorized English edition. George S. Davis, Detroit. 1893.

This work gives the physical properties and chemical reactions of 152 compounds. About one-half of this number are alkaloids. The compounds obtained from opium and cinchona are quite exhaustively treated. The author gives full directions for the preparation of the reagents which are to be used, and the temperature at which they are to be employed. This is very important, as a variation in the strength of the reagents of ten produces an entirely unlooked for result, and may lead to error in identification.

We consider this work as an excellent collection of reactions of the substances treated, and congratulate Mr. Nagelvoort upon having given to English readers an excellent translation of Prof. Flückiger's book.

We do not doubt but that this book will fill a want long felt by pharmacists and others.

The publisher has gotten the book up in splendid shape.

J. A. M.

---

OUTLINES OF PRACTICAL HISTOLOGY. A Manual for Students. By WILLIAM STIRLING, M. D., Sc. D., Brackenbury, Professor of Physiology and Histology in the Owens' College, and Professor in the Victoria University, Manchester, etc., etc. With 368 illustrations. Second edition, revised and enlarged. Pp. 419. Price, $3.00. Philadelphia: P. Blakiston, Son & Co., 1012 Walnut street. 1893.

The first edition of this work was primarily intended for the students of Owens' College, but as its value became recognized, a second edition was speedily called for. The first few chapters are devoted to a study of the microscope and accessories. There is, however, no mention of any of the American makes, which equal, if not surpass, the majority of foreign instruments. The remaining chapters of Part I. are devoted to the technique of microscopy.

We know of no place where all the various staining, fixing, hardening, and decalcifying fluids are so accurately given; also the formulæ for preparing the thousand and one different staining fluids, clearing fluids, mounting media, and injecting fluids. The chapters of Part II. are devoted to the study of the histological elements of the animal system. The manner of preparation for each tissue is described. The staining fluid which stains it to best advantage, and the characteristic of each tissue is then noted. Following the description of a tissue are exercises for advanced students. This work should be a *vade mecum* for beginners in histology, also for those who wish to refer often to the various methods. The illustrations are not as good as would be expected, and the description of the various tissues is somewhat slighted. The book, however, is worthy of careful study, but more in the laboratory and at the working-table than in the lecture-room or study.

The typographical work is nicely executed. W. C. K.

---

CHOLERA: Its Protean Aspects and its Management. By DR. G. ARCHIE STOCKWELL, F. Z. S. (Member New Sydenham Society, London). In two volumes—Vols. I. and II. "Respice, aspice, prospice." Physicians' Leisure Library. Price, twenty-five cents. Detroit, Mich.: George S. Davis. 1893.

RECENT DEVELOPMENTS IN MASSAGE—Historical, Physiological, Medical, and Surgical. By DOUGLAS GRAHAM, M. D., Boston, Mass., Fellow of the Massachusetts Medical Society; Member of Alumni Association of Jefferson Medical College, of the American Medical Association, of the British Medical Association, etc. Physicians' Leisure Library. Second edition; illustrated. Issued monthly. Price, single copies, twenty-five cents. George S. Davis, Detroit, Mich. 1893.

ELECTRO-THERAPEUTICS OF NEURASTHENIA. By W. F. ROBINSON, M. D., Physicians' Leisure Library. Issued monthly. Subscription price, $2.50; single copies, twenty-five cents. George S. Davis, Detroit, Mich. 1893.

These three books are among the latest issues of the Physicians' Leisure Library, and treat upon subjects that are of interest and importance. This kind of literature is so cheap, that we can hardly understand how it is easy to do without it. It is convenient for railway travelers, and furnishes an opportunity to beguile the hours that would otherwise hang heavily. The enterprise that supplies so much literature at so little cost, is certainly highly commendable.

A Dictionary of Medical Science. Containing a Full Explanation of the Various Subjects and Terms of Anatomy, Physiology, Medical Chemistry, Pharmacy, Pharmacology, Therapeutics, Medicine, Hygiene, Dietetics, Pathology, Surgery, Bacteriology, Ophthalmology, Otology, Laryngology, Dermatology, Gynecology, Obstetrics, Pediatrics, Medical Jurisprudence, and Dentistry, etc., etc. By Robley Dunglison, M. D., LL. D., late Professor of Institutes of Medicine in the Jefferson Medical College of Philadelphia. Edited by Richard J. Dunglison, A. M., M. D. New (twenty-first) edition, thoroughly revised, greatly enlarged and improved, with the pronunciation, accentuation, and derivation of the terms. In one magnificent imperial octavo volume of 1181 pages. Cloth, $7.00 ; leather, $8.00. Philadelphia : Lea Brothers & Co. 1893.

When Dunglison's Medical Dictionary was first published, it contained all that was essential for a dictionary to contain for students and practitioners of medicine. It is more than likely to be found, in some edition or another, on the book-shelves of nine-tenths of the medical men of the United States. It is sixty years since the first edition was put forth, during which time twenty others have been issued,—an average of one in three years. The present edition is one of the most complete that has ever been published, and is a monument to the distinguished editor, who has spared no pains to bring the work down to the present time. Up to 1869, the editions had mainly been the work of Robley Dunglison, the father, but since that time Richard J. Dunglison, the son, has assumed the entire editorship of the book.

It is a difficult task to make a single volume contain the necessities of the present as a reference word-book. The addition and multiplication of medical terms have been so rapid within the past decade, that no single dictionary has proven sufficient for the number of definitions, pronunciation, and other requirements of philological science. When, however, a dictionary is divided into two or more volumes, it becomes exceedingly cumbersome and awkward to handle. Taken all in all, we think that Dunglison's Medical Dictionary is the best single volume word-book on medicine in the English language.

---

Index-Catalogue of the Library of the Surgeon-General's Office, United States Army. Authors and subjects. Volume XIV. Sutures-Universal. Quarto, pp. 14—1016. Washington: Government Printing Office. 1893.

Another annual addition to this colossal work is lately made, and in every way it compares with its predecessors. The fourteenth volume includes 10,124 author titles, representing 6,426

volumes, and 8,850 pamphlets. It also includes 9,867 special titles of separate books and pamphlets, and 38,461 titles of articles in periodicals. The total number of titles in the index-catalogue, as far as published, is as follows: Author titles—titles, 7,453; volumes, 77,494; pamphlets, 135,656. Special titles—book titles, 151,649; journal articles, 462,165; portraits, 4,335.

This will give an idea of the stupendous nature of the undertaking, and the perseverance and industry that has been required to make the work a success. It is still in the hands of that eminent bibliographer, Dr. John S. Billings, who will most likely continue the work to a finish. While nothing is said on the subject, we presume that another volume will complete the series.

---

THE PHYSICIAN'S POCKET DAY-BOOK. Designed by C. HENRI LEONARD, M. A., M. D., Professor of Medical and Surgical Diseases of Women, and Clinical Gynecology in the Michigan College of Medicine, etc. Contains daily charges for twenty-five or fifty families weekly; has complete obstetric record for ninety-four cases, and monthly memorandum for debit and credit cash account. Price, $1.00; your name and year on the outside, in gold leaf, $1.25; name, town, state, and year, $1.50. Issued annually by the Illustrated Medical Journal Company, Detroit, Mich.

This day-book, which is also a visiting list, has been tested by fifteen years of publication and use. It is good for thirteen months, from the first day of any month in which it may be begun, and there is space for the diagnosis of each case, or for brief records of the treatment adopted, following each name. It has, among other things, the usual printed matter, such as a dose list, poisons and antidotes, urinary diseases, exanthematicæ, disinfectants, and weights and measures. The book is seven and one-half inches long, by three and one-half inches wide, so that it will hold bill-heads or currency bills without folding. It is bound in flexible cover, and weighs five ounces; hence it can be easily carried in the pocket.

---

TRANSACTIONS OF THE MEDICAL AND CHIRURGICAL SOCIETY OF THE STATE OF MARYLAND. Semi-annual Session, held at Cambridge, Md., November, 1890, and the Ninety-third Annual Session, held at Baltimore, Md., April, 1891. Octavo, paper, pp. 397. Baltimore: Griffin, Curley & Co. 1891.

We have before stated that we wished the annual volume of this society were bound in cloth, so that it might have better preservation, for its contents is always of a character well worthy of more enduring covers. In the volume before us are several attractive papers, and we may be pardoned if we speak of one or two in particular. Obstetrical Antisepsis, by J. Edwin Michael, M. D., is an intelligent setting forth of an important subject, and one that deserves full and elaborate discussion on all suitable occasions. The Revival in Physical Education and Personal Hygiene, by Edward M. Schaeffer, is another paper of great interest, and Dr. George H. Rohé's contribution, entitled, One Hundred Consecutive Cases of Labor at the Maryland Maternity, we note contains much material for thought, and deserves careful study. This society always distinguishes itself with creditable work.

---

Transactions of the Texas State Medical Association. Twenty-fourth Annual Session, held at Tyler, Texas, April 26, 27, and 28, 1892. Edited by Hamilton A. West, M. D., Secretary. Galveston: J. W. Burson Co., Printers and Publishers. 1892.

Transactions of the Texas State Medical Association. Twenty-fifth Annual Session, held at Galveston, May 2, 3, 4, and 5, 1893. Edited by Hamilton A. West, M. D., Secretary. Octavo volume, pp. 448. Galveston: Knapp Brothers, Printers and Publishers. 1893.

The lone star State has an excellent medical society that publishes its proceedings in good form. The two volumes before us, —namely, the Transactions for 1892-93, are full of excellent papers, and some of the discussions are most creditable in character. This association commits the same error that do several others, in not paying sufficient attention to the question of illustrating the subjects that are treated of in its pages. This, of course, is largely the fault of the authors, and not of the secretary or editor, and will, undoubtedly, be corrected to a great extent as time advances. Otherwise, we have nothing but words of commendation for the appearance of the books in question, for they are a credit to the society and especially to the accomplished secretary who edits the volumes.

---

Transactions of the Southern Surgical and Gynecological Association. Volume V. Fifth Session, held at Louisville, Ky., November 16, 17, and 18, 1892. Edited by William E. B. Davis, M. D., Secretary, and published by the Association. Pp. xl.—434. Philadelphia: William J. Dornan, Printer. 1893.

The fifth volume which this active association puts forth is in keeping with its predecessors, and shows also improvements in material and method. This association is doing a vast work for the profession of medicine, especially in the South and South-west, where its constituency chiefly lies. We wish that the authors might take more pains to illustrate their papers, because such a work easily becomes a text-book, and should have all of the advantages that well-illustrated articles may afford it. We hope the future volumes will present an improvement in this respect. We have nothing but praise for the character of the work done at the fifth annual meeting, as recorded in this book. It was of a very high order, and the volume deserves to have a wide circulation.

---

A Manual of Jurisprudence and Toxicology. By Henry C. Chapman, M. D., Professor of Institutes of Medicine and Medical Jurisprudence, in the Jefferson Medical College of Philadelphia; Member of the College of Physicians of Philadelphia, of the Academy of Natural Sciences of Philadelphia, of the American Philosophical Society, and of the Zoological Society of Philadelphia. With thirty-six illustrations, some of which are in colors. Price, $1.25. Philadelphia: W. B. Saunders, 913 Walnut street. 1892.

This work embraces a course of lectures delivered by the author on medical jurisprudence to the students of Jefferson Medical College during the session of 1891-92. It was prepared at the request of the students, and the author hopes that it will assist them, as well as others, in the study of this most important branch of medicine. It is a handy little book for the student, but it is not sufficiently comprehensive for the use of experts and teachers. It contains a few illustrations, some of which are very good, but their number might be increased with benefit to the reader. It is to be regretted that authors do not pay more attention to this important branch of book-making.

---

Cholera: Its Causes, Symptoms, Pathology, and Treatment. By Roberts Bartholow, M. D., LL. D., Emeritus Professor of Materia Medica, General Therapeutics, and Hygiene, in the Jefferson Medical College of Philadelphia. In one 12mo volume of 127 pages, with nine engravings. Cloth, $1.25. Philadelphia: Lea Brothers & Company. 1893.

Cholera is a disease on which the public needs much enlightenment, and the profession, likewise, will bear additional information on the subject. This present period may be considered that of the renaissance of cholera literature. In the light of recent investigations, showing the relationship of the cholera bacillus to the disease, and of the fact that it is a malady in which filth furnishes the principal avenue for its propagation, it becomes interesting to study all the newer literature on the subject. This contribution of Bartholow is an interesting one, and deserves an appropriate place amongst modern writings. The modesty of the book is commendable, and it deserves a careful reading by every physician interested in the subject.

---

TRANSACTIONS OF THE MEDICAL SOCIETY OF THE STATE OF NORTH CAROLINA. Thirty-eighth Annual Session, held at Asheville, N. C., May 26, 27, and 28, 1891. Octavo, paper, pp. 205. Wilmington, N. C.: Jackson & Bell. 1892.

The *esprit de corps* of the medical profession of the old North State is admirable. It has one of the best medical examining boards, and its medical society publishes an admirable volume of transactions that ought to be bound in cloth for permanent preservation. Dr. Karl von Ruck has an interesting paper in this volume, entitled, The Present Status of Pulmonary Tuberculosis, which is a valuable contribution to this important subject, and deserves careful study by every physician. Dr. J. W. Long publishes in this volume a syllabus of three lectures delivered before the society on Urinalysis, which is an exhaustive setting forth of the subject. The society gave Dr. Long an especial vote of thanks for his great and useful work. This volume could be improved by illustrations, which we hope to see in future numbers.

---

PROCEEDINGS OF THE PHILADELPHIA COUNTY MEDICAL SOCIETY. Volume XIII. Session of 1892. LOUIS H. ADLER, JR., M. D., editor. Philadelphia. Printed for the Society. 1893.

We are not familiar with any county society in the country that does any better work than the Philadelphia County Medical Society; nor do we know of one that keeps its records as well. Many of the papers published in this volume, together with the discussions of the same, have appeared in some of the medical journals; indeed, a few of them have been published in the JOURNAL. This society takes pains to put in type the articles of its contributors beforehand, and galley-proofs are sent out to the members and medical journals, so that the first may be prepared to discuss the paper intelligently and the second may be enabled to print it promptly. We commend this example to other medical societies who would make their work substantial and efficient.

**Transactions of the Association of American Physicians.** Eighth Session, held at Washington, D. C., May 30–31, and June 1, 1893. Volume VIII. Edited by I. Minis Hays, M. D., Recorder. Philadelphia: William J. Dornan, Printer. 1893.

This volume of society transactions appears with more promptness than that of any other national association. It is, as usual, filled with interesting papers that are handled in a scientific manner. Dr. W. Gilman Thompson's paper on A Study of Addison's Disease and of the Adrenals, will attract attention, and the paper by Dr. A. C. Abbott, on The Results of Inoculations of Milch Cows with Cultures of the Bacillus Diphtheriæ, is another one that is full of interest. But we must not specify invidiously where the whole product is so valuable. This is one of the society transaction volumes that every physician ought to possess.

---

**The Essentials of Histology, Descriptive and Practical.** For the use of Students. By E. A. Schaefer, F. R. S., Jodrell Professor of Physiology in University College, London; Editor of the histological portion of Quain's Anatomy. Third edition, revised and enlarged. Philadelphia: Lea Brothers & Co. 1892.

Schäfer's Histology has been before the profession too long to need any extended review of its strong points. This, the third edition, contains over 300 illustrations, many of which are new. All show the value of having good illustrations for students to follow in the study of microscopical science. The book is expressly designed for students, contains 298 pages, with a good index, and is cheerfully recommended not only to beginners in histology but also to advanced students in this subject.

Printing, good paper, and nicely executed cuts help to enhance the value of the book. W. C. K.

---

**Memoranda of Poisons.** By Thomas Hawkes Tanner, M. D., F. L. S. Seventh American, from the last London, edition. Revised by John J. Reese, M. D., late Professor of Medical Jurisprudence and Toxicology in the University of Pennsylvania. Philadelphia: P. Blakiston, Son & Co. 1892.

This is an old friend and a useful one. The subject of poisons is of great importance to the general practitioner, who is rarely so well equipped in this department as to be able to handle emergency cases with skill and discretion. This book serves as a ready reminder, and can be carried in the pocket or hand-bag.

Transactions of the American Gynecological Society. Volume XVII. For the year 1892. Edited by Henry Clark Coe, M. D., Secretary. Small octavo, pp. xxxix.—493. Philadelphia : William J. Dornan, Printer. 1892.

This handsome volume appears each year with a regularity and precision that is commendable. It also each year—and the present one is no exception—contains much valuable literature pertaining to the diseases of women. No specialist can afford to do without the book, and no student of gynecology should deny himself the privilege of its possession.

---

Operation Blanks. Second edition. Prepared by W. W. Keen, M. D., Professor of the Principles of Surgery in the Jefferson Medical College, Philadelphia.

This blank contains useful memoranda for physician and nurse, in making preparations for an operation. The fact that a second edition has been called for, within a short time, indicates the popularity of the method, and we especially commend it to surgeons and gynecologists who are obliged to invoke the aid of assistants in some of the preparations for their work.

